# Supplementary material for: A decade of study on the condition of western Cuban coral reefs, with low human impact
Source: PeerJ. 2023 Aug 31;11:e15953. doi: 10.7717/peerj.15953 (PMC10475277; doi:10.7717/peerj.15953)
Supplement: Supplemental Information 1 [file peerj-11-15953-s001.pdf]

A decade of study on the condition of western Cuban coral reefs, with low human impact

Hansel Caballero Aragón, Susana Perera Valderrama, Dorka Cobián Rojas, Zaimiuri Hernández Gonzáles, Juliet Gónzalez-Méndez, Elena de la Guardia Llansó.

Supplementary Materials

Table S1. Functional coefficient (FC) for each species of Guanahacabibes coral reefs, according to the criteria of González-Barrios and Álvarez-Fillip (2018).

Species	FC
Aagaricia agaricites	0.19
Acropora cervicornis	0.62
Agaricia granmahe	0.12
Agaricia lamarcki	0.08
Agaricia tenuifolia	0.26
Colpophyllia natans	0.26
Dichocoenia stockesi	0.15
Diploria laberyntiformis	0.28
Eusmilia fastigiata	0.23
Helioseris cucullata	0.03
Isophyllia sinuosa	0.07
Madracis decactis	0.28
Madracis mirabilis	0.16
Manicina areolata	0.17
Meandrina meandrites	0.12
Montastraea cavernosa	0.32
Mussa angulosa	0.09
Mycetophylia aliciae	0.06
Mycetophyllia danana	0.04
Mycetophyllia ferox	0.02
Mycetophyllia lamarckiana	0.04
Orbicella annularis	0.47
Orbicella Faveolata	0.42
Orbicella franksi	0.43
Porites astreoides	0.18
Porites divaricata	0.11
Porites furcata	0.11
Porites porites	0.22
Pseudodiploria strigosa	0.30
Scolymia lacera	0.09
Siderastrea radians	0.04
Siderastrea siderea	0.19
Stephanocoenia stockesi	0.08

Table S2. Mean (AVE) and standard deviation (SD) of living coral cover (%) per site and year. CP: Cuevas de Pedro, YE: Yemayá, VE: Veral, and VR: Verraco.

Site	Year	AVE	SD
CP	2008	23,3	6,6
CP	2010	21,2	8,9
CP	2011	21,9	7,7
CP	2012	22,1	6,3
CP	2013	22,3	6,3
CP	2014	22,0	7,9
CP	2015	21,3	3,5
CP	2016	17,8	3,6
CP	2017	17,6	6,9
YE	2008	19,7	4,5
YE	2010	22,2	7,6
YE	2011	21,6	5,7
YE	2012	22,2	7,1
YE	2013	22,5	9,6
YE	2014	19,2	5,3
YE	2015	18,2	4,6
YE	2016	15,1	4,1
YE	2017	14,6	4,1
VE	2008	16,0	8,8
VE	2010	14,1	4,1
VE	2011	14,3	4,4
VE	2012	16,7	5,1
VE	2013	14,7	6,6
VE	2014	14,3	3,4
VE	2015	16,0	6,6
VE	2016	14,9	9,2
VE	2017	14,6	8,0
VR	2008	13,0	2,9
VR	2010	14,1	4,1
VR	2011	16,0	6,9
VR	2012	14,4	4,8
VR	2013	13,9	4,9
VR	2014	14,4	6,4
VR	2015	15,9	4,7
VR	2016	13,8	6,5
VR	2017	13,8	5,5

Table S3. Pair wise test analysis for living coral cover. CP: Cuevas de Pedro, YE: Yemayá, VE: Veral, and VR: Verraco, s: site.

Groups	t	P(perm)	perms
2008, 2010	0.08	0.870	9834
2008, 2011	0.39	0.645	9826
2008, 2012	1.10	0.358	9835
2008, 2013	0.36	0.703	9822
2008, 2014	0.73	0.508	9825
2008, 2015	0.13	0.930	9839
2008, 2016	1.77	0.198	9841
2008, 2017	1.83	0.177	9829
2010, 2011	1.03	0.378	9832
2010, 2012	1.57	0.230	9847
2010, 2013	1.48	0.236	9821
2010, 2014	0.49	0.582	9830
2010, 2015	0.05	0.994	9859
2010, 2016	1.42	0.033	9833
2010, 2017	1.49	0.025	9830
2011, 2012	0.47	0.664	9849
2011, 2013	0.18	0.816	9850
2011, 2014	1.62	0.236	9834
2011, 2015	0.62	0.586	9833
2011, 2016	2.07	0.027	9830
2011, 2017	2.15	0.034	9847
2012, 2013	0.98	0.451	9814
2012, 2014	1.75	0.213	9838
2012, 2015	0.93	0.453	9814
2012, 2016	2.43	0.006	9814
2012, 2017	2.42	0.013	9836
2013, 2014	1.05	0.473	9835
2013, 2015	0.37	0.764	9843
2013, 2016	1.65	0.022	9829
2013, 2017	1.71	0.021	9835
2014, 2015	0.52	0.594	9835
2014, 2016	1.71	0.227	9843
2014, 2017	1.82	0.202	9854
2015, 2016	4.32	0.038	9838
2015, 2017	4.49	0.036	9849
2016, 2017	2.04	0.137	9795

Table S4. Mean (AVE) and standard deviation (SD) of fleshy algae index per site and year. CP: Cuevas de Pedro, YE: Yemayá, VE: Veral, and VR: Verraco.

Site	Year	AVE	SD
CP	2008	169,0	127,6
CP	2010	93,0	104,3
CP	2011	111,9	131,6
CP	2012	237,2	244,6
CP	2013	273,8	305,7
CP	2014	216,2	189,7
CP	2015	151,7	152,7
CP	2016	321,1	277,8
CP	2017	296,5	235,4
YE	2008	169,4	123,0
YE	2010	103,3	108,3
YE	2011	31,8	81,6
YE	2012	87,3	68,7
YE	2013	132,7	90,5
YE	2014	135,4	140,9
YE	2015	134,1	174,4
YE	2016	231,4	271,7
YE	2017	264,7	245,8
VE	2008	145,2	92,4
VE	2010	86,7	78,0
VE	2011	68,0	57,6
VE	2012	69,3	60,4
VE	2013	192,0	242,0
VE	2014	184,2	192,8
VE	2015	111,6	113,1
VE	2016	146,1	182,1
VE	2017	189,6	222,4
VR	2008	167,3	105,7
VR	2010	102,4	84,8
VR	2011	110,2	106,2
VR	2012	182,4	204,4
VR	2013	195,9	235,0
VR	2014	196,9	199,3
VR	2015	165,3	163,1
VR	2016	209,2	177,8
VR	2017	221,5	212,7

Table S5. Pair wise test analysis for fleshy algae index. CP: Cuevas de Pedro, YE: Yemayá, VE: Veral, and VR: Verraco, s: site.

**Within level 'CP' of factor 'site'**				**Within level 'YE' of factor 's'**				**Within level 'VE' of factor 's'**				**Within level 'VR' of factor 's'**			
Groups	t	P(perm)	perms	Groups	t	P(perm)	perms	Groups	t	P(perm)	perms	Groups	t	P(perm)	perms
2008, 2010	2.18	0.032	152	2008, 2010	0.78	0.456	67	2008, 2010	1.62	0.111	132	2008, 2010	2.18	0.032	152
2008, 2011	0.06	0.958	343	2008, 2011	3.17	0.002	196	2008, 2011	3.44	0.001	158	2008, 2011	0.06	0.958	343
2008, 2012	3.18	0.001	408	2008, 2012	4.31	0.000	346	2008, 2012	4.69	0.000	282	2008, 2012	3.18	0.001	408
2008, 2013	4.08	0.000	434	2008, 2013	1.16	0.256	1361	2008, 2013	4.72	0.000	2403	2008, 2013	4.08	0.000	434
2008, 2014	6.04	0.000	236	2008, 2014	1.53	0.127	397	2008, 2014	7.49	0.000	410	2008, 2014	6.04	0.000	236
2008, 2015	3.06	0.002	235	2008, 2015	3.24	0.002	2114	2008, 2015	1.85	0.069	198	2008, 2015	3.06	0.002	235
2008, 2016	5.07	0.000	1339	2008, 2016	4.81	0.000	1804	2008, 2016	2.63	0.008	676	2008, 2016	5.07	0.000	1339
2008, 2017	5.49	0.000	1632	2008, 2017	5.62	0.000	1977	2008, 2017	7.01	0.000	365	2008, 2017	5.49	0.000	1632
2010, 2011	1.77	0.080	334	2010, 2011	2.40	0.019	199	2010, 2011	2.07	0.044	159	2010, 2011	1.77	0.080	334
2010, 2012	3.92	0.000	416	2010, 2012	3.32	0.001	345	2010, 2012	3.22	0.002	258	2010, 2012	3.92	0.000	416
2010, 2013	4.81	0.000	430	2010, 2013	0.64	0.532	954	2010, 2013	5.21	0.000	2413	2010, 2013	4.81	0.000	430
2010, 2014	7.74	0.000	252	2010, 2014	2.25	0.022	407	2010, 2014	8.75	0.000	426	2010, 2014	7.74	0.000	252
2010, 2015	4.47	0.000	240	2010, 2015	3.71	0.001	2124	2010, 2015	2.96	0.004	199	2010, 2015	4.47	0.000	240
2010, 2016	6.32	0.000	1375	2010, 2016	5.19	0.000	1848	2010, 2016	3.69	0.001	689	2010, 2016	6.32	0.000	1375
2010, 2017	6.82	0.000	1700	2010, 2017	6.02	0.000	2002	2010, 2017	7.86	0.000	382	2010, 2017	6.82	0.000	1700
2011, 2012	3.03	0.003	421	2011, 2012	0.29	0.777	330	2011, 2012	0.82	0.416	286	2011, 2012	3.03	0.003	421
2011, 2013	3.90	0.000	827	2011, 2013	1.03	0.320	967	2011, 2013	5.83	0.000	2507	2011, 2013	3.90	0.000	827
2011, 2014	5.29	0.000	490	2011, 2014	4.38	0.000	424	2011, 2014	9.52	0.000	476	2011, 2014	5.29	0.000	490
2011, 2015	2.72	0.008	485	2011, 2015	4.94	0.000	2275	2011, 2015	4.23	0.000	223	2011, 2015	2.72	0.008	485
2011, 2016	4.44	0.000	1421	2011, 2016	6.12	0.000	2005	2011, 2016	4.56	0.000	808	2011, 2016	4.44	0.000	1421
2011, 2017	4.94	0.000	1741	2011, 2017	6.87	0.000	2135	2011, 2017	8.20	0.000	435	2011, 2017	4.94	0.000	1741
2012, 2013	0.72	0.470	524	2012, 2013	1.40	0.175	1312	2012, 2013	5.92	0.000	2471	2012, 2013	0.72	0.470	524
2012, 2014	0.18	0.862	416	2012, 2014	5.58	0.000	418	2012, 2014	10.56	0.000	461	2012, 2014	0.18	0.862	416
2012, 2015	1.38	0.172	427	2012, 2015	5.97	0.000	1268	2012, 2015	4.94	0.000	211	2012, 2015	1.38	0.172	427
2012, 2016	0.21	0.831	2192	2012, 2016	7.55	0.000	1954	2012, 2016	5.45	0.000	773	2012, 2016	0.21	0.831	2192
2012, 2017	0.15	0.882	2505	2012, 2017	8.18	0.000	2168	2012, 2017	8.93	0.000	424	2012, 2017	0.15	0.882	2505
2013, 2014	1.10	0.280	424	2013, 2014	2.34	0.018	1487	2013, 2014	1.36	0.180	2199	2013, 2014	1.10	0.280	424
2013, 2015	2.25	0.028	448	2013, 2015	3.61	0.001	2659	2013, 2015	3.69	0.000	2385	2013, 2015	2.25	0.028	448
2013, 2016	0.45	0.651	2245	2013, 2016	4.29	0.000	2352	2013, 2016	2.31	0.024	2678	2013, 2016	0.45	0.651	2245
2013, 2017	0.68	0.499	2572	2013, 2017	5.56	0.000	2816	2013, 2017	0.09	0.927	1792	2013, 2017	0.68	0.499	2572
2014, 2015	1.99	0.051	272	2014, 2015	2.33	0.024	2195	2014, 2015	4.25	0.000	448	2014, 2015	1.99	0.051	272
2014, 2016	0.60	0.548	1395	2014, 2016	3.89	0.001	1804	2014, 2016	2.86	0.006	884	2014, 2016	0.60	0.548	1395
2014, 2017	0.52	0.611	1681	2014, 2017	4.97	0.000	1992	2014, 2017	1.65	0.106	355	2014, 2017	0.52	0.611	1681
2015, 2016	1.98	0.053	1532	2015, 2016	1.34	0.183	2130	2015, 2016	0.67	0.510	892	2015, 2016	1.98	0.053	1532
2015, 2017	2.19	0.030	1792	2015, 2017	2.19	0.034	4200	2015, 2017	4.42	0.000	407	2015, 2017	2.19	0.030	1792
2016, 2017	0.14	0.887	3059	2016, 2017	0.57	0.574	3647	2016, 2017	3.22	0.003	998	2016, 2017	0.14	0.887	3059

Table S6. Mean of coral species richness (per sample unit) per site and year. CP: Cuevas de Pedro, YE: Yemayá, VE: Veral, and VR: Verraco.

Year	Site	AVE	SD
2008	CP	5,67	1,54
2008	YE	5,93	2,19
2008	VE	5,29	1,61
2008	VR	4,60	1,06
2010	CP	6,20	1,70
2010	YE	5,93	1,58
2010	VE	5,07	1,44
2010	VR	4,20	0,94
2011	CP	6,40	0,91
2011	YE	5,40	0,99
2011	VE	4,93	1,75
2011	VR	5,07	1,39
2012	CP	5,63	1,50
2012	YE	5,87	1,81
2012	VE	6,20	2,21
2012	VR	4,60	1,24
2013	CP	5,91	1,54
2013	YE	6,33	1,91
2013	VE	4,53	1,77
2013	VR	4,94	1,26
2014	CP	5,80	1,57
2014	YE	5,53	1,30
2014	VE	4,73	1,03
2014	VR	4,80	1,78
2015	CP	6,07	1,53
2015	YE	4,69	1,62
2015	VE	4,81	1,56
2015	VR	5,00	2,30
2016	CP	5,20	1,42
2016	YE	5,09	2,12
2016	VE	5,73	1,58
2016	VR	5,46	1,85
2017	CP	6,60	1,40
2017	YE	6,07	1,49
2017	VE	6,27	1,83
2017	VR	6,38	2,29

Table S7. Pair wise test analysis for coral richness. CP: Cuevas de Pedro, YE: Yemayá, VE: Veral, and VR: Verraco, s: site.

**Within level 'CP' of factor 's'**				**Within level 'YE' of factor 's'**				**Within level 'VE' of factor 's'**				**Within level 'VR' of factor 's'**			
Groups	t	P(perm)	perms	Groups	t	P(perm)	perms	Groups	t	P(perm	perms	Groups	t	P(perm	perms
2008, 2010	1.00	0.384	17	2008, 2010	0.10	1.000	17	2008, 2010	0.91	0.424	31	2008, 2010	0.62	0.642	12
2008, 2011	1.28	0.266	13	2008, 2011	0.40	0.786	17	2008, 2011	1.05	0.356	31	2008, 2011	0.42	0.785	13
2008, 2012	0.16	0.901	30	2008, 2012	0.09	1.000	17	2008, 2012	1.43	0.180	37	2008, 2012	0.80	0.531	11
2008, 2013	0.66	0.576	34	2008, 2013	0.17	0.939	21	2008, 2013	1.15	0.299	17	2008, 2013	0.84	0.414	27
2008, 2014	0.12	1.000	15	2008, 2014	0.71	0.572	15	2008, 2014	1.15	0.313	29	2008, 2014	0.45	0.732	18
2008, 2015	0.36	0.814	14	2008, 2015	1.90	0.068	38	2008, 2015	0.64	0.592	32	2008, 2015	0.10	1.000	17
2008, 2016	0.12	1.000	16	2008, 2016	1.64	0.123	35	2008, 2016	0.78	0.515	17	2008, 2016	1.40	0.184	30
2008, 2017	2.19	0.052	16	2008, 2017	0.32	0.777	30	2008, 2017	1.99	0.065	36	2008, 2017	4.25	0.001	30
2010, 2011	Negative			2010, 2011	0.38	0.806	13	2010, 2011	0.13	1.000	13	2010, 2011	1.01	0.391	12
2010, 2012	0.90	0.442	16	2010, 2012	Negative			2010, 2012	2.15	0.047	19	2010, 2012	0.17	1.000	10
2010, 2013	0.50	0.681	34	2010, 2013	0.29	0.849	18	2010, 2013	0.28	0.820	30	2010, 2013	1.49	0.172	26
2010, 2014	0.89	0.438	18	2010, 2014	0.78	0.525	15	2010, 2014	0.15	1.000	12	2010, 2014	0.91	0.438	16
2010, 2015	0.68	0.578	16	2010, 2015	2.20	0.036	32	2010, 2015	0.25	0.811	29	2010, 2015	0.50	0.701	17
2010, 2016	1.09	0.338	16	2010, 2016	1.96	0.076	27	2010, 2016	1.70	0.128	16	2010, 2016	1.91	0.090	29
2010, 2017	1.02	0.371	16	2010, 2017	0.29	0.802	26	2010, 2017	2.88	0.010	18	2010, 2017	4.98	0.000	17
2011, 2012	1.17	0.327	13	2011, 2012	0.34	0.822	15	2011, 2012	2.27	0.040	18	2011, 2012	1.19	0.310	13
2011, 2013	0.61	0.607	27	2011, 2013	0.60	0.619	19	2011, 2013	0.16	0.909	29	2011, 2013	0.38	0.799	31
2011, 2014	1.14	0.330	13	2011, 2014	0.41	0.786	12	2011, 2014	Negative			2011, 2014	0.11	1.000	18
2011, 2015	0.89	0.465	14	2011, 2015	1.93	0.067	30	2011, 2015	0.38	0.725	28	2011, 2015	0.19	0.929	20
2011, 2016	1.39	0.224	14	2011, 2016	1.73	0.095	27	2011, 2016	1.84	0.099	14	2011, 2016	1.00	0.357	30
2011, 2017	1.33	0.255	12	2011, 2017	0.10	1.000	26	2011, 2017	3.03	0.009	20	2011, 2017	3.60	0.001	29
2012, 2013	0.52	0.659	31	2012, 2013	0.27	0.855	19	2012, 2013	2.36	0.030	38	2012, 2013	1.68	0.123	26
2012, 2014	0.03	1.000	30	2012, 2014	0.70	0.573	16	2012, 2014	2.43	0.026	17	2012, 2014	1.04	0.358	17
2012, 2015	0.22	0.903	28	2012, 2015	2.01	0.059	34	2012, 2015	1.94	0.072	36	2012, 2015	0.60	0.624	19
2012, 2016	0.28	0.817	30	2012, 2016	1.76	0.108	33	2012, 2016	0.76	0.514	19	2012, 2016	2.07	0.063	28
2012, 2017	2.15	0.046	30	2012, 2017	0.26	0.817	27	2012, 2017	0.28	0.856	20	2012, 2017	5.27	0.000	28
2013, 2014	0.53	0.657	33	2013, 2014	0.90	0.421	17	2013, 2014	0.17	0.901	29	2013, 2014	0.19	0.918	34
2013, 2015	0.27	0.818	33	2013, 2015	2.07	0.048	42	2013, 2015	0.52	0.659	32	2013, 2015	0.48	0.632	39
2013, 2016	0.78	0.452	32	2013, 2016	1.79	0.091	36	2013, 2016	1.89	0.075	36	2013, 2016	0.76	0.489	30
2013, 2017	1.76	0.098	33	2013, 2017	0.51	0.611	36	2013, 2017	3.04	0.008	20	2013, 2017	3.50	0.003	30
2014, 2015	0.24	0.901	16	2014, 2015	1.58	0.124	30	2014, 2015	0.42	0.691	25	2014, 2015	0.25	0.869	22
2014, 2016	0.23	0.911	15	2014, 2016	1.42	0.189	25	2014, 2016	2.05	0.069	15	2014, 2016	0.75	0.480	36
2014, 2017	2.07	0.064	16	2014, 2017	0.51	0.680	24	2014, 2017	3.34	0.004	19	2014, 2017	2.76	0.013	34
2015, 2016	0.48	0.718	15	2015, 2016	0.02	1.000	26	2015, 2016	1.41	0.184	30	2015, 2016	0.92	0.375	38
2015, 2017	1.88	0.092	17	2015, 2017	1.99	0.060	30	2015, 2017	2.58	0.022	36	2015, 2017	2.67	0.018	39
2016, 2017	2.27	0.042	17	2016, 2017	1.79	0.092	27	2016, 2017	1.21	0.270	17	2016, 2017	2.06	0.057	30

Table S8. Relative abundance (%) of coral species per site and year. CP: Cuevas de Pedro, YE: Yemayá, VE: Veral, and VR: Verraco.

Year	2008	2008	2008	2008	2010	2010	2010	2010	2011	2011	2011	2011	2012	2012	2012	2012	2013	2013	2013	2013	2014	2014	2014	2014	2015	2015	2015	2015	2016	2016	2016	2016	2017	2017	2017	2017
Site	CP	YE	VE	VR	CP	YE	VE	VR	CP	YE	VE	VR	CP	YE	VE	VR	CP	YE	VE	VR	CP	YE	VE	VR	CP	YE	VE	VR	CP	YE	VE	VR	CP	YE	VE	VR
Agaricia agaricites	12	19	6	16	20	20	17	24	16	32	28	16	25	19	28	20	26	14	38	33	31	27	33	22	20	34	29	18	27	18	23	17	28	25	22	19
Siderastrea siderea	10	14	18	39	17	15	18	27	16	20	19	26	21	25	14	9	17	20	18	16	12	16	17	25	9	8	13	30	17	20	19	26	14	20	20	26
Orbicella faveolata	34	17	29	10	20	22	24	15	20	18	10	17	17	13	13	24	19	24	13	11	10	14	14	12	20	10	8	6	7	19	17	11	5	9	12	8
Porites astreoides	12	6	17	17	18	17	12	10	10	4	17	22	16	17	14	25	13	6	12	22	18	18	11	19	14	21	13	15	13	17	8	16	15	18	9	12
Stephanocoenia intersepta	3	8	9	1	6	6	9	2	1	6	3	1	1	5	6	5	0	7	3	2	4	9	9	2	2	15	4	6	4	4	8	3	4	5	8	4
Porites porites	1	3	1	0	1	1	1	4	2	2	2	3	3	2	2	2	5	1	6	3	5	2	6	12	8	0	5	4	8	1	0	10	8	1	2	8
Montastraea cavernosa	11	3	2	9	2	3	2	2	4	2	5	3	3	3	3	4	5	4	1	2	2	1	0	2	5	2	6	3	4	3	3	2	3	2	2	3
Porites furcata	1	0	0	0	3	1	4	3	7	1	0	1	4	1	2	1	3	1	3	6	1	1	1	3	2	0	3	3	3	2	1	1	6	3	3	3
Orbicella annularis	1	0	0	0	0	1	2	2	5	3	5	2	2	2	1	0	4	1	4	3	3	1	1	1	5	1	2	1	3	2	3	3	2	1	2	1
Colpophyllia natans	4	11	8	1	0	2	1	0	4	0	2	3	2	1	1	2	1	4	1	1	1	2	0	1	1	0	0	1	2	1	1	1	2	1	1	1
Agaricia lamarcki	2	2	1	1	2	6	3	1	0	1	0	0	1	2	1	4	1	5	1	0	1	1	4	0	0	1	2	1	1	3	4	1	2	3	3	1
Millepora alcycornis	0	0	1	0	0	1	2	2	1	1	0	0	0	2	1	1	0	1	0	0	1	1	0	1	2	4	5	3	3	4	3	1	3	4	6	2
Orbicella franksi	1	7	2	0	4	0	2	0	4	1	4	3	1	3	1	2	1	3	0	0	0	1	1	0	2	1	2	2	0	1	1	1	0	1	0	1
Meandrina meandrites	3	1	1	0	0	2	2	1	1	4	0	0	1	1	3	0	1	4	0	0	1	2	0	0	1	0	4	2	1	0	1	1	1	1	1	1
Madracis decactis	2	1	0	0	1	0	0	1	1	1	0	0	2	0	3	2	0	0	0	1	1	0	0	0	1	0	2	2	1	0	1	1	1	1	1	3
Acropora cervicornis	0	2	0	0	1	2	0	0	2	0	0	0	1	0	1	0	1	1	0	1	3	2	0	0	4	0	0	0	3	0	0	0	2	0	0	0
Pseudodiploria strigosa	0	0	1	1	2	1	0	0	1	0	0	1	0	1	2	0	0	1	0	0	0	0	0	1	1	0	2	1	0	3	0	0	1	2	0	0
Diploria laberinthyiformis	0	0	3	1	0	1	0	1	2	1	1	1	0	1	0	0	0	1	0	0	1	0	0	0	0	0	0	0	1	0	1	0	1	0	1	0
Agaricia tenufolia	0	0	0	0	1	0	0	1	1	0	0	0	1	1	1	0	1	0	0	1	3	0	0	0	2	0	0	0	0	0	1	1	1	0	1	1
Eusmillia fastigiata	0	0	0	0	0	1	0	0	1	0	1	0	0	1	0	0	0	1	0	0	0	0	0	0	2	0	0	1	0	0	2	0	1	1	2	1
Manicina areolata	0	0	0	1	0	0	0	0	0	0	2	0	0	0	0	0	0	0	1	0	0	0	2	0	0	0	0	1	0	0	1	2	1	0	1	2
Mycetophyllia danana	1	0	1	0	1	0	2	1	0	0	0	0	0	0	1	0	1	0	1	1	1	0	0	0	0	0	0	1	0	0	1	0	0	0	1	0
Porites divaricata	0	2	0	0	0	0	0	0	1	0	1	1	0	0	0	0	0	1	0	0	0	0	0	0	0	0	0	0	0	0	0	3	0	1	0	2
Dichocoenia stockesi	1	0	0	0	0	0	0	0	1	1	0	0	0	0	1	0	0	1	0	0	1	0	0	0	0	0	0	0	1	0	1	0	1	0	1	0
Mycetophyllia lamarckiana	0	0	0	0	1	0	1	0	1	1	2	0	0	0	1	1	0	0	0	0	0	0	0	1	0	0	0	0	1	0	0	0	1	0	0	0
Mussa angulosa	0	0	0	0	1	0	0	0	1	0	0	0	0	1	0	0	0	0	0	0	0	2	1	0	0	0	0	0	0	1	1	0	0	1	1	1
Siderastrea radians	0	0	0	0	1	0	0	0	0	0	0	0	0	0	0	0	2	0	0	0	0	0	0	0	0	0	0	1	1	0	0	0	1	1	0	1
Madracis mirabilis	0	1	0	0	0	1	0	0	0	1	0	0	0	0	0	0	0	0	0	0	1	0	0	0	1	0	1	0	0	0	1	0	0	0	1	0
Helioseris cucullata	1	0	0	0	0	0	0	0	0	1	0	0	0	0	1	0	0	0	0	0	0	0	0	0	0	0	0	1	1	0	0	0	1	0	0	0
Isophyllia rigida	0	0	0	0	0	0	0	1	0	0	0	0	0	0	1	1	0	0	0	1	0	0	0	0	0	0	0	0	0	0	0	0	1	0	0	0
Mycetophyllia aliciae	1	1	0	0	0	0	0	0	0	1	0	0	0	0	0	0	0	1	0	0	0	0	0	0	0	0	0	0	0	0	0	0	0	0	0	0
Scolymia lacera	0	0	0	0	0	0	0	0	0	0	0	0	0	0	0	0	0	0	0	0	0	0	0	0	0	0	0	0	0	1	0	0	0	1	0	0
Mycetophyllia ferox	1	1	0	0	0	0	0	0	0	0	0	0	0	0	0	0	0	0	0	0	0	0	0	0	0	0	0	0	0	0	0	0	0	0	0	0
Agaricia granmahe	0	0	0	0	0	0	0	0	0	0	0	0	0	0	0	0	0	0	0	0	0	1	0	0	0	0	0	0	0	0	0	0	0	0	0	0

Table S9. Pair wise test analysis for coral relative abundance. CP: Cuevas de Pedro, YE: Yemayá, VE: Veral, and VR: Verraco, s: site.

Within level 'CP' of factor 's'				Within level 'YE' of factor 's'				Within level 'VE' of factor 's'				Within level 'VR' of factor 's'			
Groups	t	P(perm)	perms	Groups	t	P(perm)	perms	Groups	t	P(perm)	perms	Groups	t	P(perm)	perms
2008, 2010	3.13	0.000	9943	2008, 2010	1.50	0.028	9934	2008, 2010	1.97	0.000	9944	2008, 2010	1.99	0.001	9944
2008, 2011	3.03	0.000	9950	2008, 2011	1.76	0.002	9937	2008, 2011	2.31	0.000	9935	2008, 2011	1.54	0.054	9940
2008, 2012	3.11	0.000	9947	2008, 2012	1.47	0.044	9936	2008, 2012	2.16	0.000	9940	2008, 2012	2.41	0.001	9951
2008, 2013	3.08	0.000	9949	2008, 2013	1.31	0.110	9945	2008, 2013	2.96	0.000	9950	2008, 2013	2.59	0.000	9943
2008, 2014	2.62	0.000	9943	2008, 2014	1.28	0.131	9944	2008, 2014	2.64	0.000	9945	2008, 2014	1.82	0.010	9954
2008, 2015	2.55	0.000	9944	2008, 2015	1.67	0.006	9951	2008, 2015	2.45	0.000	9937	2008, 2015	1.55	0.022	9934
2008, 2016	2.82	0.000	9941	2008, 2016	1.54	0.023	9945	2008, 2016	2.11	0.000	9943	2008, 2016	2.20	0.000	9942
2008, 2017	3.38	0.000	9938	2008, 2017	1.94	0.000	9950	2008, 2017	2.24	0.000	9947	2008, 2017	2.01	0.000	9923
2010, 2011	1.81	0.002	9952	2010, 2011	1.44	0.047	9953	2010, 2011	1.81	0.005	9933	2010, 2011	1.36	0.090	9944
2010, 2012	1.49	0.044	9949	2010, 2012	1.19	0.227	9953	2010, 2012	1.33	0.111	9934	2010, 2012	1.70	0.011	9943
2010, 2013	2.04	0.000	9939	2010, 2013	1.24	0.175	9936	2010, 2013	2.35	0.000	9948	2010, 2013	2.09	0.000	9926
2010, 2014	1.53	0.042	9949	2010, 2014	1.14	0.266	9950	2010, 2014	1.85	0.002	9946	2010, 2014	0.88	0.606	9938
2010, 2015	1.64	0.010	9934	2010, 2015	1.58	0.017	9944	2010, 2015	1.72	0.008	9948	2010, 2015	1.32	0.095	9943
2010, 2016	1.81	0.003	9955	2010, 2016	0.69	0.804	9953	2010, 2016	1.32	0.118	9953	2010, 2016	0.95	0.509	9948
2010, 2017	2.27	0.000	9942	2010, 2017	1.31	0.143	9955	2010, 2017	1.61	0.017	9967	2010, 2017	0.86	0.655	9942
2011, 2012	1.36	0.078	9945	2011, 2012	1.66	0.016	9943	2011, 2012	0.99	0.467	9939	2011, 2012	1.71	0.012	9955
2011, 2013	1.33	0.106	9964	2011, 2013	1.82	0.004	9950	2011, 2013	1.55	0.032	9949	2011, 2013	1.91	0.003	9950
2011, 2014	1.55	0.032	9945	2011, 2014	1.55	0.023	9948	2011, 2014	1.57	0.032	9947	2011, 2014	1.32	0.142	9935
2011, 2015	1.06	0.370	9953	2011, 2015	2.01	0.000	9957	2011, 2015	0.98	0.464	9946	2011, 2015	1.47	0.054	9948
2011, 2016	1.23	0.175	9939	2011, 2016	1.65	0.021	9940	2011, 2016	1.48	0.056	9945	2011, 2016	1.50	0.058	9955
2011, 2017	1.81	0.003	9937	2011, 2017	1.96	0.002	9948	2011, 2017	1.80	0.004	9940	2011, 2017	1.55	0.042	9947
2012, 2013	1.08	0.327	9947	2012, 2013	1.32	0.110	9936	2012, 2013	1.60	0.023	9954	2012, 2013	2.09	0.001	9940
2012, 2014	0.89	0.575	9958	2012, 2014	0.78	0.712	9949	2012, 2014	1.13	0.288	9971	2012, 2014	2.05	0.004	9952
2012, 2015	1.28	0.131	9950	2012, 2015	1.74	0.005	9940	2012, 2015	1.16	0.243	9950	2012, 2015	2.05	0.001	9940
2012, 2016	0.91	0.577	9927	2012, 2016	0.78	0.693	9944	2012, 2016	1.05	0.387	9954	2012, 2016	2.11	0.001	9933
2012, 2017	1.38	0.069	9954	2012, 2017	1.03	0.390	9953	2012, 2017	1.42	0.061	9949	2012, 2017	2.25	0.000	9928
2013, 2014	1.09	0.324	9946	2013, 2014	1.61	0.023	9941	2013, 2014	0.85	0.629	9956	2013, 2014	1.82	0.009	9947
2013, 2015	0.85	0.662	9939	2013, 2015	2.28	0.000	9955	2013, 2015	1.09	0.334	9949	2013, 2015	1.61	0.011	9961
2013, 2016	1.02	0.416	9957	2013, 2016	1.30	0.137	9942	2013, 2016	1.49	0.055	9953	2013, 2016	1.95	0.001	9962
2013, 2017	1.70	0.006	9947	2013, 2017	2.07	0.001	9951	2013, 2017	1.61	0.018	9960	2013, 2017	2.05	0.000	9943
2014, 2015	0.61	0.862	9939	2014, 2015	1.41	0.061	9945	2014, 2015	1.21	0.203	9950	2014, 2015	1.08	0.322	9933
2014, 2016	0.57	0.894	9949	2014, 2016	1.26	0.164	9956	2014, 2016	1.30	0.141	9942	2014, 2016	0.56	0.887	9953
2014, 2017	0.96	0.488	9946	2014, 2017	1.16	0.240	9940	2014, 2017	1.55	0.027	9952	2014, 2017	0.53	0.910	9941
2015, 2016	0.72	0.807	9951	2015, 2016	1.78	0.007	9957	2015, 2016	1.32	0.117	9943	2015, 2016	1.16	0.230	9944
2015, 2017	1.31	0.109	9950	2015, 2017	1.77	0.004	9950	2015, 2017	1.43	0.067	9956	2015, 2017	1.08	0.320	9934
2016, 2017	0.60	0.890	9944	2016, 2017	0.83	0.593	9934	2016, 2017	0.60	0.879	9954	2016, 2017	0.47	0.940	9880

Table S10. Relative abundance (%) of coral traits groups per site and year. CP: Cuevas de Pedro, YE: Yemayá, VE: Veral, and VR: Verraco.

Year	2008	2008	2008	2008	2010	2010	2010	2010	2011	2011	2011	2011	2012	2012	2012	2012
Site	CP	YE	VE	VR	CP	YE	VE	VR	CP	YE	VE	VR	CP	YE	VE	VR
Foliose-digitate species	3	7	1	0	5	2	5	9	10	5	2	4	9	2	7	4
Framework-building branching corals	0	2	0	0	1	2	0	0	2	0	0	0	1	0	1	0
Massive reef framework-building corals	35	23	31	10	24	23	28	18	29	22	19	23	19	18	15	25
Non-framework-building corals	30	31	25	36	44	44	35	39	29	39	49	38	42	41	47	51
Secundary Massive reef framework-building corals	32	37	43	54	27	29	32	34	30	33	30	35	28	38	30	20

Year	2013	2013	2013	2013	2014	2014	2014	2014	2015	2015	2015	2015	2016	2016	2016	2016	2017	2017	2017	2017
Site	CP	YE	VE	VR	CP	YE	VE	VR	CP	YE	VE	VR	CP	YE	VE	VR	CP	YE	VE	VR
Foliose-digitate species	8	2	9	9	7	3	7	14	11	1	11	9	12	3	2	15	15	5	7	16
Framework-building branching corals	1	1	0	1	3	2	0	0	4	0	0	0	3	0	0	0	2	0	0	0
Massive reef framework-building corals	24	28	16	13	14	17	16	12	28	13	13	9	10	23	21	15	6	11	15	10
Non-framework-building corals	43	27	52	57	54	49	50	41	39	60	47	39	45	41	42	37	50	51	42	39
Secundary Massive reef framework-building corals	24	41	23	20	21	30	26	32	18	26	30	43	31	32	34	33	26	32	36	35

Table S11. Pair wise test analysis for relative abundance of coral traits groups. CP: Cuevas de Pedro, YE: Yemayá, VE: Veral, and VR: Verraco, s: site.

**Within level 'CP' of factor 's'**				**Within level 'YE' of factor 's'**				**Within level 'VE' of factor 's'**				**Within level 'VR' of factor 's'**			
Groups	t	P(perm)	perms	Groups	t	P(perm)	perms	Groups	t	P(perm)	perms	Groups	t	P(perm)	perms
2008, 2010	2.85	0.000	9950	2008, 2010	1.62	0.037	9961	2008, 2010	3.26	0.000	9961	2008, 2010	3.27	0.000	9959
2008, 2011	3.43	0.000	9954	2008, 2011	1.81	0.011	9952	2008, 2011	2.71	0.000	9958	2008, 2011	2.34	0.003	9958
2008, 2012	3.31	0.000	9943	2008, 2012	1.65	0.038	9954	2008, 2012	2.71	0.000	9947	2008, 2012	3.07	0.000	9943
2008, 2013	3.23	0.000	9950	2008, 2013	1.03	0.382	9959	2008, 2013	3.21	0.000	9950	2008, 2013	3.19	0.000	9953
2008, 2014	2.93	0.000	9945	2008, 2014	1.56	0.068	9947	2008, 2014	2.98	0.000	9968	2008, 2014	2.62	0.000	9949
2008, 2015	2.98	0.000	9943	2008, 2015	2.43	0.001	9952	2008, 2015	3.30	0.000	9944	2008, 2015	1.21	0.225	9956
2008, 2016	3.41	0.000	9958	2008, 2016	1.69	0.029	9945	2008, 2016	2.33	0.001	9962	2008, 2016	2.73	0.000	9947
2008, 2017	3.84	0.000	9958	2008, 2017	2.36	0.001	9938	2008, 2017	2.23	0.002	9948	2008, 2017	2.71	0.000	9964
2010, 2011	1.49	0.076	9966	2010, 2011	1.70	0.030	9954	2010, 2011	1.58	0.068	9958	2010, 2011	2.01	0.009	9970
2010, 2012	1.04	0.369	9957	2010, 2012	1.66	0.059	9972	2010, 2012	1.67	0.044	9963	2010, 2012	2.06	0.005	9962
2010, 2013	0.61	0.777	9949	2010, 2013	2.01	0.009	9958	2010, 2013	1.95	0.017	9964	2010, 2013	2.41	0.001	9961
2010, 2014	1.30	0.165	9952	2010, 2014	1.09	0.339	9960	2010, 2014	1.88	0.019	9949	2010, 2014	1.45	0.103	9964
2010, 2015	1.21	0.222	9951	2010, 2015	1.95	0.016	9955	2010, 2015	1.91	0.011	9952	2010, 2015	2.20	0.002	9958
2010, 2016	1.78	0.013	9957	2010, 2016	1.03	0.405	9953	2010, 2016	0.92	0.488	9950	2010, 2016	1.17	0.279	9957
2010, 2017	2.32	0.001	9963	2010, 2017	1.67	0.047	9958	2010, 2017	1.48	0.085	9934	2010, 2017	2.28	0.001	9962
2011, 2012	1.75	0.023	9963	2011, 2012	1.97	0.009	9961	2011, 2012	0.44	0.890	9952	2011, 2012	1.57	0.067	9955
2011, 2013	1.68	0.040	9947	2011, 2013	2.00	0.008	9952	2011, 2013	0.83	0.597	9959	2011, 2013	2.23	0.001	9960
2011, 2014	2.06	0.007	9946	2011, 2014	2.17	0.002	9967	2011, 2014	0.49	0.860	9953	2011, 2014	1.44	0.121	9969
2011, 2015	1.33	0.143	9963	2011, 2015	3.08	0.000	9954	2011, 2015	1.16	0.281	9958	2011, 2015	1.48	0.073	9958
2011, 2016	2.08	0.004	9956	2011, 2016	0.14	0.912	9957	2011, 2016	1.10	0.323	9953	2011, 2016	1.65	0.056	9954
2011, 2017	3.21	0.000	9957	2011, 2017	2.17	0.002	9946	2011, 2017	1.24	0.229	9964	2011, 2017	1.99	0.007	9958
2012, 2013	0.78	0.623	9971	2012, 2013	1.44	0.111	9961	2012, 2013	0.65	0.761	9970	2012, 2013	1.38	0.123	9954
2012, 2014	0.71	0.712	9961	2012, 2014	1.31	0.190	9960	2012, 2014	Negative			2012, 2014	1.92	0.011	9955
2012, 2015	1.60	0.042	9960	2012, 2015	2.23	0.010	9962	2012, 2015	1.14	0.294	9948	2012, 2015	1.96	0.005	9952
2012, 2016	0.66	0.749	9953	2012, 2016	1.66	0.056	9952	2012, 2016	0.84	0.581	9947	2012, 2016	2.06	0.002	9946
2012, 2017	1.36	0.135	9951	2012, 2017	1.77	0.041	9948	2012, 2017	0.51	0.855	9940	2012, 2017	2.53	0.000	9962
2013, 2014	0.86	0.556	9954	2013, 2014	2.10	0.007	9959	2013, 2014	Negative			2013, 2014	1.80	0.019	9963
2013, 2015	1.14	0.275	9928	2013, 2015	3.14	0.000	9975	2013, 2015	1.18	0.257	9960	2013, 2015	1.81	0.010	9960
2013, 2016	1.50	0.073	9955	2013, 2016	1.79	0.027	9950	2013, 2016	1.43	0.111	9971	2013, 2016	1.91	0.007	9955
2013, 2017	1.99	0.007	9961	2013, 2017	2.89	0.000	9950	2013, 2017	1.29	0.178	9951	2013, 2017	2.06	0.004	9945
2014, 2015	1.36	0.128	9966	2014, 2015	1.19	0.260	9951	2014, 2015	1.12	0.315	9964	2014, 2015	1.37	0.123	9955
2014, 2016	0.77	0.642	9940	2014, 2016	1.70	0.035	9951	2014, 2016	1.29	0.174	9953	2014, 2016	0.36	0.896	9958
2014, 2017	0.83	0.572	9962	2014, 2017	1.35	0.152	9954	2014, 2017	1.15	0.281	9946	2014, 2017	0.68	0.725	9959
2015, 2016	1.91	0.004	9952	2015, 2016	2.39	0.001	9947	2015, 2016	1.81	0.023	9960	2015, 2016	1.53	0.066	9954
2015, 2017	2.56	0.000	9946	2015, 2017	1.82	0.029	9954	2015, 2017	1.47	0.075	9957	2015, 2017	1.28	0.170	9959
2016, 2017	1.13	0.306	9949	2016, 2017	1.49	0.099	9916	2016, 2017	0.64	0.750	9943	2016, 2017	0.8187	0.592	9921

Table S12. Mean and standard deviation of the reef functional index per site and year. CP: Cuevas de Pedro, YE: Yemayá, VE: Veral, and VR: Verraco.

Year	Site	AVE	SD
2008	CP	0,50	0,04
2008	YE	0,43	0,05
2008	VE	0,44	0,07
2008	VR	0,40	0,04
2010	CP	0,40	0,03
2010	YE	0,40	0,04
2010	VE	0,37	0,02
2010	VR	0,34	0,04
2011	CP	0,41	0,02
2011	YE	0,38	0,03
2011	VE	0,37	0,04
2011	VR	0,39	0,03
2012	CP	0,40	0,03
2012	YE	0,40	0,04
2012	VE	0,39	0,03
2012	VR	0,37	0,05
2013	CP	0,41	0,03
2013	YE	0,41	0,04
2013	VE	0,38	0,03
2013	VR	0,38	0,05
2014	CP	0,40	0,04
2014	YE	0,41	0,03
2014	VE	0,37	0,03
2014	VR	0,37	0,04
2015	CP	0,43	0,03
2015	YE	0,40	0,04
2015	VE	0,35	0,04
2015	VR	0,37	0,06
2016	CP	0,38	0,04
2016	YE	0,38	0,04
2016	VE	0,38	0,03
2016	VR	0,37	0,03
2017	CP	0,40	0,03
2017	YE	0,38	0,02
2017	VE	0,39	0,03
2017	VR	0,39	0,02

Table S13. Pair wise test analysis for the reef functional index. CP: Cuevas de Pedro, YE: Yemayá, VE: Veral, and VR: Verraco, s: site.

**Within level 'CP' of factor 's'**				**Within level 'YE' of factor 's'**				**Within level 'VE' of factor 's'**				**Within level 'VR' of factor 's'**			
Groups	t	P(perm)	perms	Groups	t	P(perm)	perms	Groups	t	P(perm)	perms	Groups	t	P(perm)	perms
2008, 2010	6.95	0.000	60	2008, 2010	1.57	0.136	46	2008, 2010	3.71	0.001	120	2008, 2010	4.54	0.000	49
2008, 2011	6.53	0.000	51	2008, 2011	3.24	0.003	50	2008, 2011	3.42	0.002	128	2008, 2011	0.98	0.366	38
2008, 2012	6.80	0.000	110	2008, 2012	1.83	0.082	48	2008, 2012	2.60	0.017	111	2008, 2012	1.98	0.066	46
2008, 2013	7.19	0.000	113	2008, 2013	0.88	0.399	49	2008, 2013	3.38	0.002	62	2008, 2013	1.49	0.157	86
2008, 2014	6.47	0.000	62	2008, 2014	1.23	0.240	42	2008, 2014	3.49	0.002	118	2008, 2014	2.08	0.055	40
2008, 2015	5.21	0.000	49	2008, 2015	1.87	0.080	90	2008, 2015	4.36	0.000	132	2008, 2015	1.76	0.087	48
2008, 2016	7.88	0.000	68	2008, 2016	2.57	0.014	93	2008, 2016	2.93	0.008	117	2008, 2016	2.16	0.045	70
2008, 2017	7.52	0.000	59	2008, 2017	3.07	0.007	83	2008, 2017	2.84	0.008	117	2008, 2017	1.20	0.274	28
2010, 2011	1.04	0.339	24	2010, 2011	2.12	0.052	32	2010, 2011	0.06	1.000	30	2010, 2011	4.10	0.000	40
2010, 2012	0.09	0.953	59	2010, 2012	0.35	0.760	33	2010, 2012	1.86	0.085	27	2010, 2012	2.08	0.054	42
2010, 2013	0.91	0.389	61	2010, 2013	0.73	0.499	37	2010, 2013	0.77	0.464	54	2010, 2013	2.63	0.012	89
2010, 2014	0.00	1.000	31	2010, 2014	0.64	0.563	29	2010, 2014	0.13	0.950	28	2010, 2014	2.61	0.017	36
2010, 2015	2.11	0.054	30	2010, 2015	0.35	0.769	67	2010, 2015	1.39	0.183	56	2010, 2015	1.83	0.081	45
2010, 2016	2.06	0.050	32	2010, 2016	1.59	0.124	69	2010, 2016	1.35	0.217	26	2010, 2016	2.54	0.020	66
2010, 2017	0.59	0.605	26	2010, 2017	1.94	0.074	56	2010, 2017	1.31	0.226	28	2010, 2017	4.42	0.000	36
2011, 2012	0.89	0.414	54	2011, 2012	1.69	0.113	32	2011, 2012	1.42	0.177	37	2011, 2012	1.29	0.220	39
2011, 2013	0.14	0.900	57	2011, 2013	2.66	0.017	37	2011, 2013	0.56	0.592	65	2011, 2013	0.72	0.509	77
2011, 2014	0.91	0.405	30	2011, 2014	3.26	0.003	30	2011, 2014	0.05	1.000	35	2011, 2014	1.31	0.219	34
2011, 2015	1.28	0.247	24	2011, 2015	1.73	0.097	62	2011, 2015	1.19	0.251	69	2011, 2015	1.12	0.302	42
2011, 2016	3.02	0.003	32	2011, 2016	0.00	1.000	59	2011, 2016	0.99	0.346	34	2011, 2016	1.44	0.175	58
2011, 2017	1.72	0.105	26	2011, 2017	0.37	0.766	45	2011, 2017	0.99	0.347	38	2011, 2017	0.17	0.889	49
2012, 2013	0.78	0.444	65	2012, 2013	1.04	0.337	36	2012, 2013	1.02	0.328	62	2012, 2013	0.49	0.643	92
2012, 2014	0.08	0.959	64	2012, 2014	1.02	0.344	30	2012, 2014	1.49	0.168	31	2012, 2014	0.17	0.899	42
2012, 2015	1.94	0.064	60	2012, 2015	0.01	1.000	66	2012, 2015	2.77	0.009	72	2012, 2015	Negative		
2012, 2016	2.10	0.046	69	2012, 2016	1.27	0.229	68	2012, 2016	0.52	0.654	30	2012, 2016	0.09	0.968	37
2012, 2017	0.66	0.547	56	2012, 2017	1.48	0.156	52	2012, 2017	0.43	0.702	32	2012, 2017	1.23	0.243	64
2013, 2014	0.83	0.436	63	2013, 2014	0.25	0.842	32	2013, 2014	0.55	0.595	59	2013, 2014	0.37	0.743	81
2013, 2015	1.42	0.178	63	2013, 2015	1.05	0.310	75	2013, 2015	1.94	0.056	64	2013, 2015	0.45	0.684	92
2013, 2016	3.10	0.003	73	2013, 2016	2.06	0.053	77	2013, 2016	0.52	0.606	58	2013, 2016	0.44	0.691	76
2013, 2017	1.57	0.126	57	2013, 2017	2.50	0.018	64	2013, 2017	0.54	0.591	65	2013, 2017	0.62	0.589	68
2014, 2015	1.89	0.080	33	2014, 2015	1.03	0.326	58	2014, 2015	1.34	0.208	64	2014, 2015	0.15	0.918	43
2014, 2016	1.90	0.078	35	2014, 2016	2.35	0.030	64	2014, 2016	1.03	0.348	29	2014, 2016	0.09	0.956	64
2014, 2017	0.52	0.644	31	2014, 2017	3.25	0.003	49	2014, 2017	1.02	0.346	32	2014, 2017	1.30	0.217	55
2015, 2016	3.79	0.000	37	2015, 2016	1.31	0.204	73	2015, 2016	2.37	0.023	64	2015, 2016	0.08	0.974	39
2015, 2017	2.74	0.011	28	2015, 2017	1.52	0.141	54	2015, 2017	2.30	0.029	65	2015, 2017	1.04	0.342	69
2016, 2017	1.62	0.137	31	2016, 2017	0.26	0.806	56	2016, 2017	0.06	1.000	31	2016, 2017	1.51	0.167	24

Table S14. Mean (AVE) and standard deviation (SD) of abiotic variables per site and year. CP: Cuevas de Pedro, YE: Yemayá, VE: Veral, and VR: Verraco.

					Years					
**Variable**	**Sites**	**2007**	**2009**	**2010**	**2011**	**2012**	**2013**	**2014**	**2015**	**2016**
AVE WAVE (m)	CP	0.44	0.33	0.47	0.59	0.42	0.68	0.44	0.23	0.74
	YE	0.41	0.31	0.43	0.55	0.42	0.64	0.45	0.21	0.74
	VE	0.31	0.29	0.43	0.42	0.26	0.40	0.24	0.17	0.48
	VR	0.44	0.33	0.47	0.59	0.42	0.68	0.44	0.23	0.74
SD WAVE (m)	CP	0.32	0.32	0.29	0.21	0.43	0.40	0.21	0.29	0.56
	YE	0.32	0.32	0.29	0.21	0.43	0.40	0.21	0.29	0.56
	VE	0.25	0.33	0.42	0.15	0.33	0.23	0.10	0.18	0.44
	VR	0.32	0.32	0.29	0.21	0.43	0.40	0.21	0.29	0.56
AVE NSST (°C)	CP	28.0	28.1	27.6	28.3	27.9	27.9	28.3	28.6	28.5
	YE	28.0	28.1	27.6	28.3	27.9	27.9	28.3	28.6	28.5
	VE	28.0	28.1	27.6	28.3	27.9	27.9	28.3	28.6	28.5
	VR	28.0	28.0	27.8	28.1	27.9	28.0	28.2	28.8	28.6
SD NSST (°C)	CP	1.3	1.8	1.7	1.8	1.3	1.0	1.5	1.4	1.4
	YE	1.3	1.8	1.7	1.8	1.3	1.0	1.5	1.4	1.4
	VE	1.3	1.8	1.7	1.8	1.3	1.0	1.5	1.4	1.4
	VR	1.2	1.7	1.6	1.6	1.3	0.9	1.4	1.3	1.3
AVE DHW (°C)	CP	1.2	4.8	0.8	0.0	1.2	0.0	0.0	3.7	3.5
	YE	1.1	3.8	0.8	0.0	0.9	0.0	0.0	3.5	1.9
	VE	0.9	3.3	1.6	0.0	0.0	0.0	0.0	3.6	3.3
	VR	0.9	2.8	0.9	0.0	0.9	0.0	0.0	4.1	3.6
SD DHW (°C)	CP	0.0	3.3	1.7	0.0	0.7	0.0	0.0	2.3	1.4
	YE	0.3	3.3	0.7	0.0	0.6	0.0	0.0	1.9	1.5
	VE	0.3	2.7	0.7	0.0	0.6	0.0	0.0	1.8	1.2
	VR	0.3	2.2	0.8	0.0	0.5	0.0	0.0	1.4	0.6
AVE POC (mol m^{-3})	CP	41.3	37.1	39.6	35.2	41.2	46.5	37.0	37.7	44.5
	YE	41.9	37.1	39.6	35.2	39.6	46.5	37.0	37.1	44.5
	VE	38.7	36.0	35.8	33.2	38.0	42.8	34.4	35.1	46.4
	VR	40.6	36.7	35.8	35.1	32.6	43.9	34.1	35.0	45.9
SD POC (mol m^{-3})	CP	7.8	8.8	6.9	7.0	13.7	13.2	7.2	6.0	9.6
	YE	7.2	8.8	6.9	7.0	10.8	13.2	7.2	6.2	9.6
	VE	7.4	8.2	8.4	6.7	12.1	14.4	9.0	6.3	12.7
	VR	9.2	8.2	5.5	5.9	8.1	10.9	9.1	5.8	16.1
AVE CHL1 (mg m^{-3})	CP	0.13	0.11	0.12	0.11	0.12	0.15	0.11	0.11	0.14
	YE	0.12	0.13	0.12	0.12	0.12	0.13	0.11	0.12	0.14
	VE	0.13	0.12	0.12	0.12	0.13	0.15	0.12	0.12	0.15
	VR	0.14	0.14	0.11	0.15	0.12	0.14	0.11	0.12	0.16
SD CHL1 (mg m^{-3})	CP	0.03	0.03	0.03	0.03	0.04	0.05	0.03	0.03	0.04
	YE	0.02	0.02	0.03	0.02	0.01	0.03	0.03	0.02	0.04
	VE	0.02	0.02	0.03	0.02	0.03	0.05	0.02	0.02	0.05
	VR	0.05	0.05	0.04	0.09	0.05	0.07	0.03	0.02	0.06
AVE KD490 (m^{-1})	CP	0.029	0.028	0.030	0.027	0.029	0.034	0.028	0.028	0.033
	YE	0.030	0.027	0.029	0.025	0.028	0.034	0.028	0.027	0.032
	VE	0.028	0.027	0.026	0.024	0.026	0.031	0.026	0.025	0.033
	VR	0.030	0.029	0.026	0.027	0.027	0.031	0.027	0.027	0.034
SD KD490 (m^{-1})	CP	0.004	0.005	0.004	0.005	0.007	0.007	0.005	0.003	0.006
	YE	0.005	0.004	0.004	0.004	0.006	0.008	0.006	0.003	0.006
	VE	0.004	0.005	0.005	0.004	0.007	0.009	0.006	0.002	0.008
	VR	0.005	0.005	0.004	0.007	0.008	0.010	0.006	0.003	0.009
AVE PAR (E m^{-2} day^{-1})	CP	41.6	43.8	41.7	42.0	42.1	41.3	42.5	42.8	42.2
	YE	41.1	43.2	41.0	41.0	41.0	41.0	41.6	41.4	40.6
	VE	40.5	42.4	40.5	40.2	39.3	40.6	40.6	40.2	40.6
	VR	41.1	42.2	41.3	40.0	39.9	40.9	42.4	41.3	41.5
SD PAR (E m^{-2} day^{-1})	CP	6.7	8.5	8.3	9.0	6.7	8.3	7.5	8.7	8.1
	YE	6.8	8.3	8.1	8.4	6.0	8.1	7.6	8.3	8.1
	VE	6.7	8.1	7.1	7.8	4.9	7.6	6.7	7.8	8.3
	VR	6.9	9.0	7.2	8.3	5.0	7.3	7.5	8.6	6.7

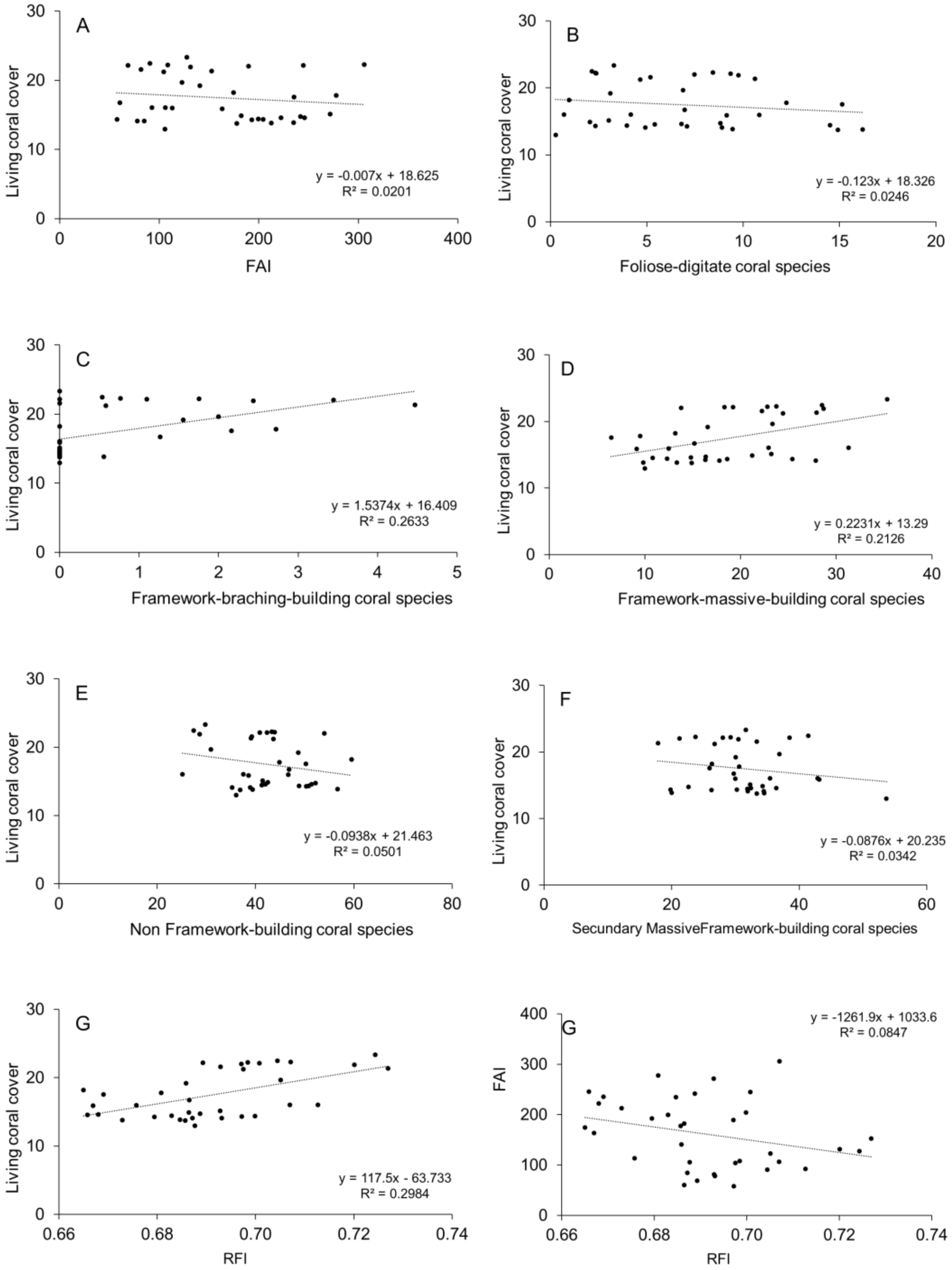

Figure S1. Correlation between biological variables of the Guanahacabibes study. FAI: fleshy algae index, RFI: reef functional index.

References

González-Barrios FJ, Álvarez-Filip L. 2018. A framework for measuring coral species-specific contribution to reef functioning in the Caribbean. *Ecological Indicators 95*: 877–886 https://doi.org/10.1016/j.ecoli nd.2018.08.038
